# Supplementary material for: Presynaptic stochasticity improves energy efficiency and helps alleviate the stability-plasticity dilemma
Source: eLife. 2021 Oct 18;10:e69884. doi: 10.7554/eLife.69884 (PMC8716105; doi:10.7554/eLife.69884)
Supplement: Source data 1. [file elife-69884-data1.zip › lifelong-mlp_bar_final-acc_all.pdf]

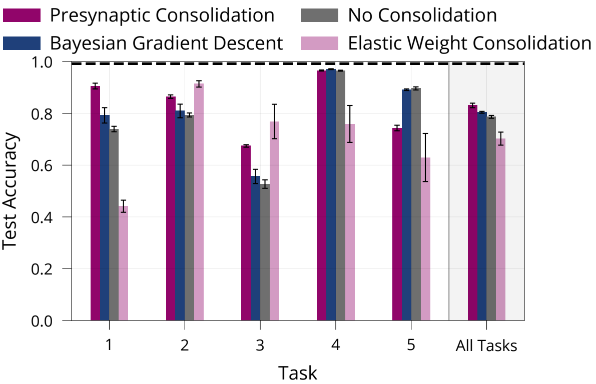

Presynaptic Consolidation
No Consolidation
Bayesian Gradient Descent
Elastic Weight Consolidation
Test Accuracy
1.0
0.8
0.6
0.4
0.2
0.0
1
2
3
4
5
All Tasks
Task
