# Supplementary figures and images for: Presynaptic stochasticity improves energy efficiency and helps alleviate the stability-plasticity dilemma
Source: eLife. 2021 Oct 18;10:e69884. doi: 10.7554/eLife.69884 (PMC8716105; doi:10.7554/eLife.69884)

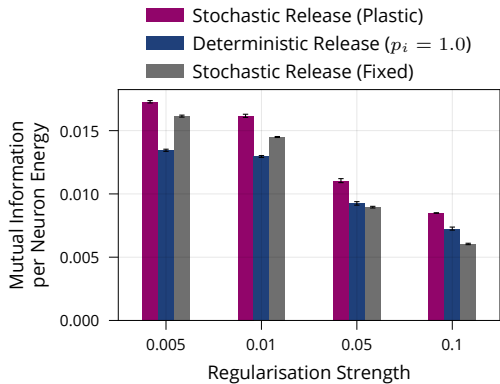

Supplement: Source data 1. [file elife-69884-data1.zip › energy-mlp_bar_information-energy-l2_neuron.pdf]

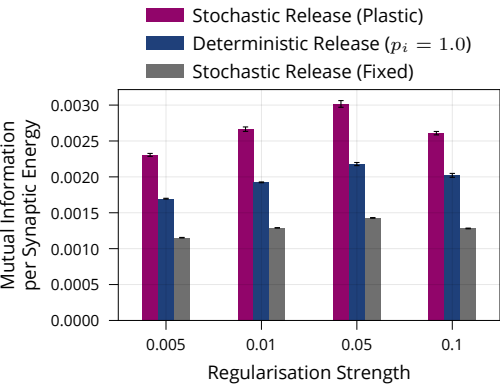

Supplement: Source data 1. [file elife-69884-data1.zip › energy-mlp_bar_information-energy-l2_synapse.pdf]

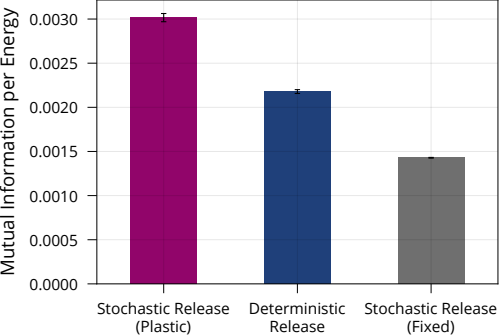

Supplement: Source data 1. [file elife-69884-data1.zip › energy-mlp_bar_information-energy.pdf]

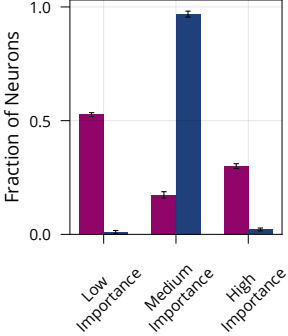

Supplement: Source data 1. [file elife-69884-data1.zip › energy-mlp_bar_neuron-activity.pdf]

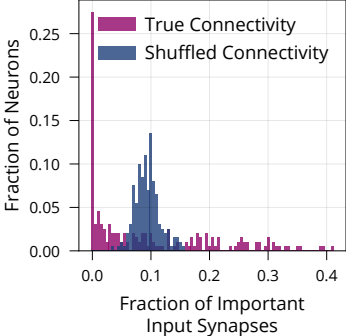

Supplement: Source data 1. [file elife-69884-data1.zip › energy-mlp_hist_neuron-connectivity.pdf]

True Connectivity Shuffled Connectivity

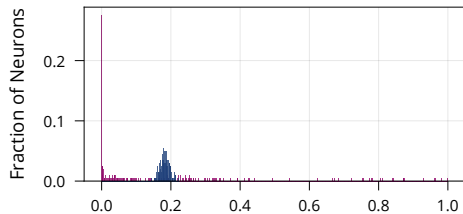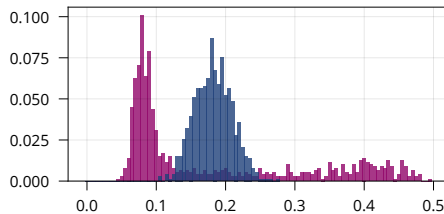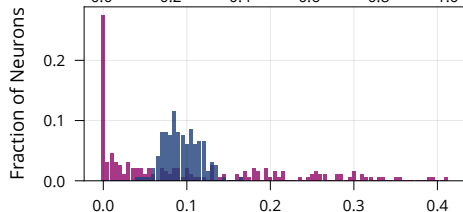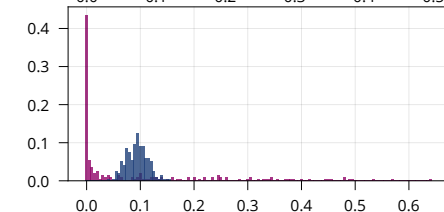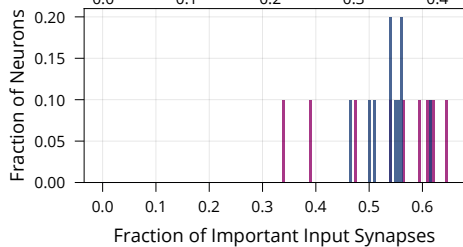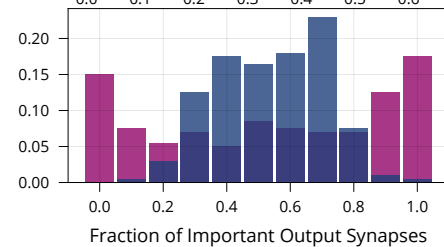

Supplement: Source data 1. [file elife-69884-data1.zip › energy-mlp_hist_neuron-connectivity_all.pdf]

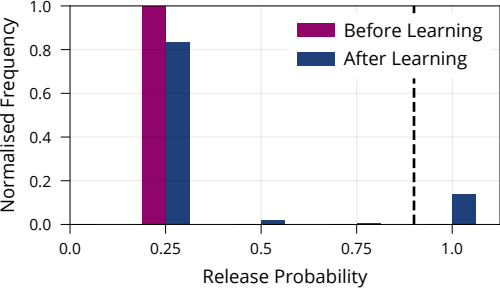

Supplement: Source data 1. [file elife-69884-data1.zip › energy-mlp_hist_release-probabilities.pdf]

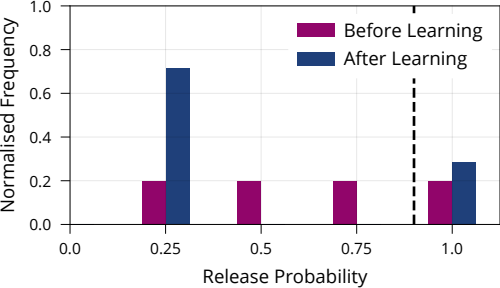

Supplement: Source data 1. [file elife-69884-data1.zip › energy-mlp_hist_release-probabilities_random-init.pdf]

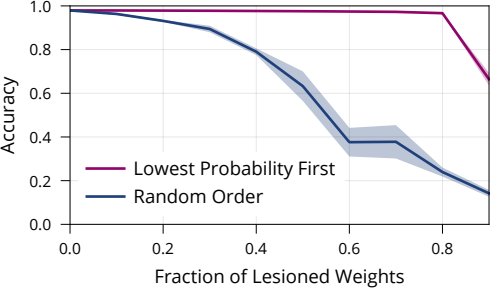

Supplement: Source data 1. [file elife-69884-data1.zip › energy-mlp_line_importance-lesion.pdf]

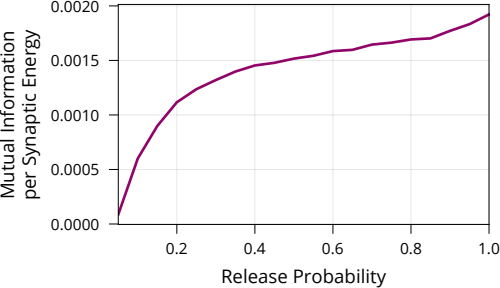

Supplement: Source data 1. [file elife-69884-data1.zip › energy-mlp_line_prob-max-info.pdf]

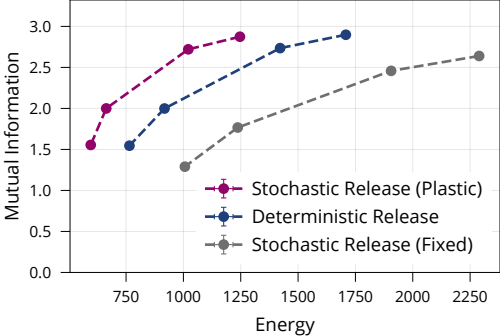

Supplement: Source data 1. [file elife-69884-data1.zip › energy-mlp_scatter_information-energy-l2.pdf]

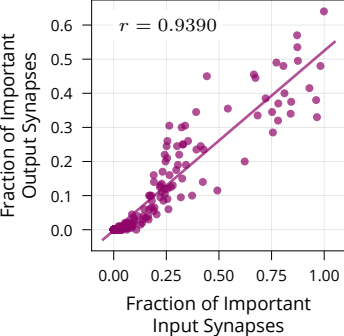

Supplement: Source data 1. [file elife-69884-data1.zip › energy-mlp_scatter_neuron-connectivity.pdf]

● True Connectivity

● Shuffled Connectivity

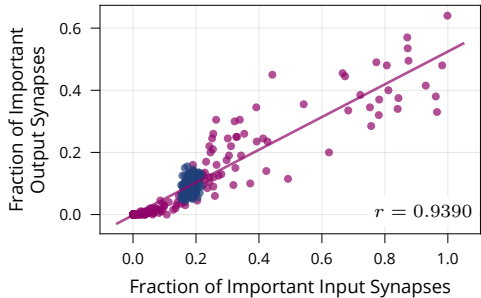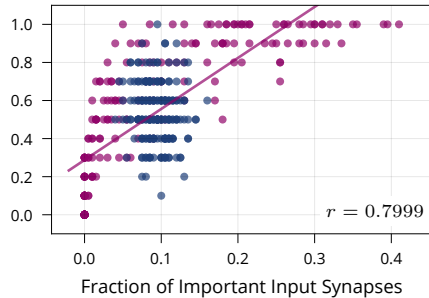

Supplement: Source data 1. [file elife-69884-data1.zip › energy-mlp_scatter_neuron-connectivity_all.pdf]

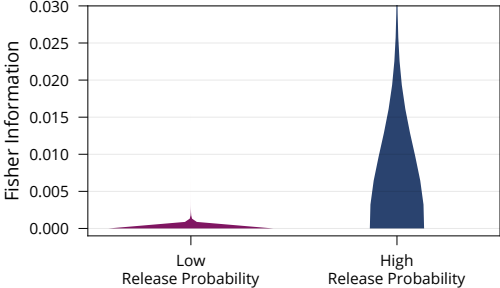

Supplement: Source data 1. [file elife-69884-data1.zip › energy-mlp_violin_fisher.pdf]

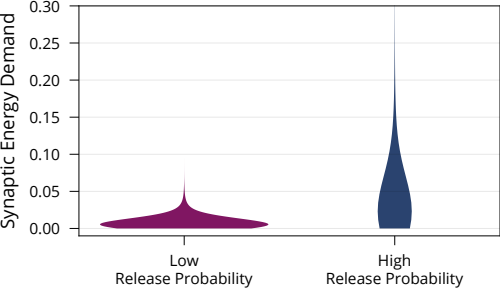

Supplement: Source data 1. [file elife-69884-data1.zip › energy-mlp_violin_release-probs-weights.pdf]

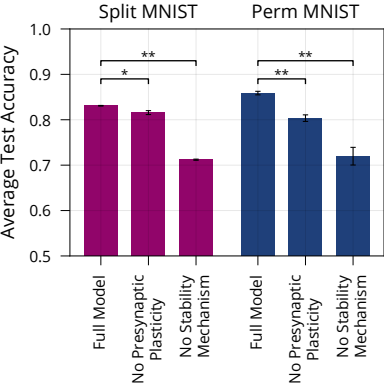

Supplement: Source data 1. [file elife-69884-data1.zip › lifelong-mlp_bar_ablation.pdf]

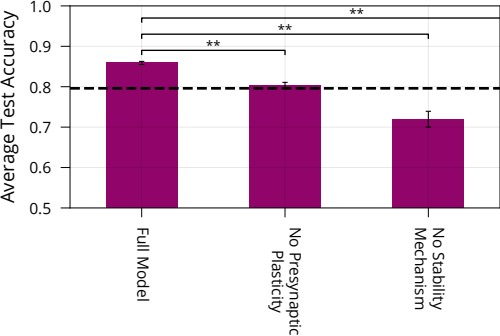

Supplement: Source data 1. [file elife-69884-data1.zip › lifelong-mlp_bar_ablation_perm-mnist.pdf]

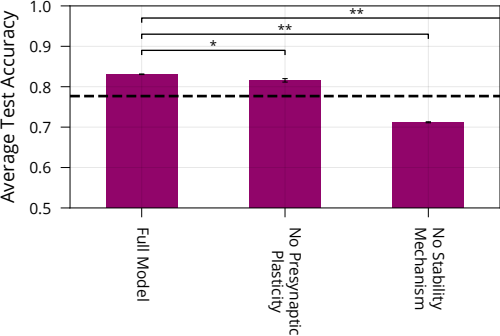

Supplement: Source data 1. [file elife-69884-data1.zip › lifelong-mlp_bar_ablation_split-mnist.pdf]

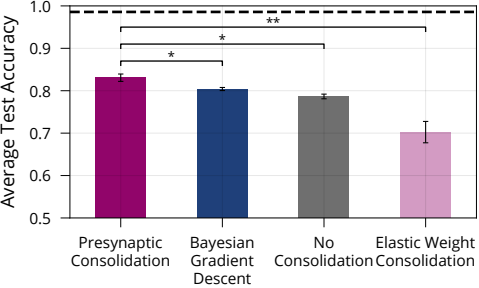

Supplement: Source data 1. [file elife-69884-data1.zip › lifelong-mlp_bar_final-acc.pdf]

— Mean Release Probability  
— Ratio of Frozen Release Probabilities

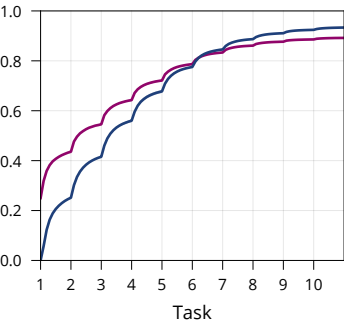

Supplement: Source data 1. [file elife-69884-data1.zip › lifelong-mlp_line_time-probs_perm-mnist.pdf]

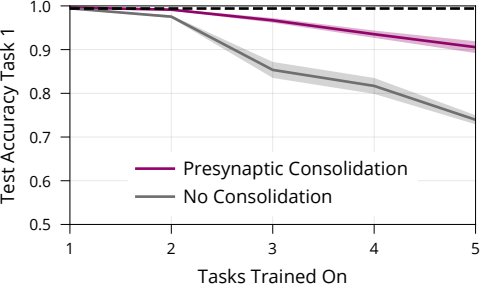

Supplement: Source data 1. [file elife-69884-data1.zip › lifelong-mlp_line_time-task1-acc.pdf]

Average Accuracy

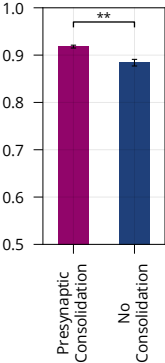

Supplement: Source data 1. [file elife-69884-data1.zip › lifelong-perceptron_bar_final-acc.pdf]

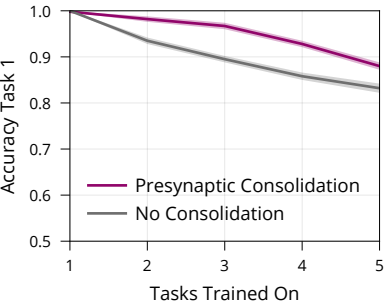

Supplement: Source data 1. [file elife-69884-data1.zip › lifelong-perceptron_line_time-task1-acc.pdf]
